# Viable Properties of Natural Rubber/Halloysite Nanotubes Composites Affected by Various Silanes

**DOI:** 10.3390/polym15010029

**Published:** 2022-12-21

**Authors:** Nabil Hayeemasae, Abdulhakim Masa, Nadras Othman, Indra Surya

**Affiliations:** 1 Research Unit of Advanced Elastomeric Materials and Innovations for BCG Economy (AEMI), Faculty of Science and Technology, Prince of Songkla University, Pattani Campus, Pattani 94000, Thailand; 2 Department of Rubber Technology and Polymer Science, Faculty of Science and Technology, Prince of Songkla University, Pattani Campus, Pattani 94000, Thailand; 3 Rubber Engineering & Technology Program, International College, Prince of Songkla University, Hat Yai, Songkhla 90110, Thailand; 4 School of Materials and Mineral Resources Engineering, Universiti Sains Malaysia, Engineering Campus, Penang 14300, Malaysia; 5 Department of Chemical Engineering, Faculty of Engineering, Universitas Sumatera Utara, Medan 20155, Indonesia

**Keywords:** natural rubber, halloysite nanotubes, silane, wide-angle X-Ray scattering

## Abstract

Natural rubber (NR) is incompatible with hydrophilic additives like halloysite nanotubes (HNT) due to their different polarity. The silane coupling agent is the ideal component to include in such a compound to solve this problem. Many types of silane are available for polymer composites depending on their functionalities. This work aimed to tune it to the composite based on NR and HNT. Four different silanes, namely Bis[3- (Triethoxysilyl)Propyl]Tetrasulfide (TESPT), 3-Aminopropyl triethoxysilane (APTES), N-[3-(Trimethoxysilyl)Propyl] Ethylenediamine (AEAPTMS), and Vinyltrimethoxysilane (VTMS) were used. Here, the mechanical properties were used to assess the properties, paying close attention to how their reinforcement influenced their crystallization behavior after stretching. It was revealed that adding silane coupling agents greatly improved the composites’ modulus, tensile strength, and tear strength. From the overall findings, AEAPTMS was viable for NR/HNT composites. This was in direct agreement with the interactions between NR and HNT that silanes had encouraged. The findings from stress-strain curves describing the crystallization of the composites are in good agreement with the findings from synchrotron wide-angle X-ray scattering (WAXS). The corresponding silanes have substantially aided the strain-induced crystallization (SIC) of composites.

## 1. Introduction

Generally, the manufacturing of rubber products requires the addition of additives to improve their properties [1,2,3]. Among commercial fillers, carbon black and precipitated silica is the most important reinforcing fillers, and these are extensively used when high strength is essential. However, for some applications where cost and processability are of greatest concern, the use of non- or semi-reinforcing fillers such as clay, talc, and calcium carbonate (CaCO3), as well as other fillers from renewable resources and waste materials, is highly recommended. The application of nanoparticle-size fillers in rubbers has received much attention regardless of organic and inorganic nanofillers [4,5]. This is because nanoparticle fillers have unique characteristics such as shape, particle size, and high surface area, which can interact well with polymers. In current times, the selection of new nanofillers is increasing growth. One of them is a fibrous nanoparticle filler or tubular material [6,7]. Because such fillers possess a higher surface area and aspect ratio than those conventional fillers, this allows greater adhesion to the polymer surface.

Halloysite nanotubes, or HNT, are an interesting tubular nanofiller. It has high mechanical strength, thermal stability, and biocompatibility [8,9,10]. HNT has gained much attention for reinforcing natural and synthetic rubbers. HNT is an economically viable material that can be mined from the consequent deposit as a raw mineral. The chemical structure of HNT is similar to clay. The limitation in filler distribution has made scientists encounter problems with mixing the HNT in non-polar rubber, especially in natural rubber (NR). Moreover, the HNT can interact with each other, forming filler–filler interactions, and causing agglomeration when adding a certain amount [11,12]. This factor gives poor performance. Therefore, efficient dispersion and increasing the compatibility of HNT and NR is an interesting approach.

Currently, a variety of research studies are conducted on increasing the compatibility of HNT in non-polar rubbers. One of the methods has been the addition of a compatibilizer or coupling agent. There have been many types of compatibilizers and/or coupling agents used for NR/HNT composites [13,14,15]. Over the years, silane coupling agents have been extensively used and studied. Because of this, the use of silane is known to have practical applications. Additionally, the theory underlying such advancement in composites has also received widespread recognition. Many types of silane have been grafted onto HNT when preparing polymer composites. Masa and Hayeemasae [14] used various types of silane in the ENR/HNT composites. It was reported that sulfur-based silane had given optimum properties. This is because the contained sulfur provided an extra crosslinking to the vulcanizates, resulting in an enhancement in the overall characteristics of the composites. The treatment of silane with (3-aminopropyl) triethoxysilane (APTES) was carried out by Yuan et al. [16]. They discovered that the grafting was successfully attached to the silanol groups of HNT together with the condensation between hydrolyzed APTES and the post-grafted APTES. Additionally, it was discovered that the treatment significantly increased the grafting ratio and enhanced the loading of hydrolyzed APTES into the HNT lumen. In addition to this, Vinyl trimethoxysilane (VTMS) was employed in nano-alumina-filled silicone rubber by He et al. [17]. VTMS enabled the generation of a reaction with the hydroxyl and carboxylic acid groups on the nano-alumina surface during the curing process, improving the filler–rubber interaction while decreasing the filler–filler interactions. The silane coupling agent Bis[3-(triethoxysilyl)propyl]tetrasulfide (TESPT) was also mechanically mixed with styrene butadiene rubber (SBR)/clay [18], TESPT served as the molecular bridge between the SBR matrix and clay filler and strengthened the interfacial interaction.

Because of the variable performance of silanes, different silanes may have given unique characteristics to the composite. This study was interested in focusing on the use of various silanes, namely Bis[3-(Triethoxysilyl)Propyl]Tetrasulfide (TESPT), 3-Aminopropyl triethoxysilane (APTES), N-[3-(Trimethoxysilyl)Propyl] Ethylenediamine (AEAPTMS), and Vinyltrimethoxysilane (VTMS). Each silane type contains different functionalities that enable the establishment of rubber–filler interactions with the NR and HNT. The properties were evaluated from the Payne effect and mechanical strength. This work also reported on the correlation between mechanical properties with structural changes analyzed by a special technique. The enhancement of rubber–filler interactions of the composite was not only verified from their mechanical and dynamic properties but was also evaluated from the development of crystallization during stretching conditions. This investigation will advance scientific knowledge of how silanes might affect the characteristics of NR/HNT composites and also serve as a valuable resource for creating rubber goods based on NR/HNT composites.

## 2. Experimental Setup

### 2.1. Materials

**Figure 1:**
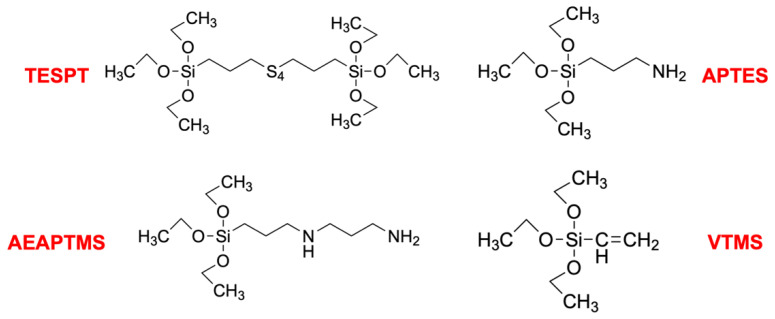
Type of silane coupling agents used for NR/HNT composites.

The NR used in this experiment was Ribbed Smoked Sheet 3 (RSS3), which was fabricated by Khok Phan Tan Rubber Fund Cooperative Ltd. (Pattani, Thailand). The silanes namely Bis[3-(Triethoxysilyl)Propyl]Tetrasulfide (TESPT; M_W_ = 538.95 g/mol), 3-Aminopropyl Triethoxysilane (APTES; M_W_ = 221.37 g/mol), N-[3-(Trimethoxysilyl)Propyl] Ethylenediamine (AEAPTMS; M_W_ = 222.36 g/mol) and Vinyltrimethoxysilane (VTMS; M_W_ = 148.23 g/mol) were purchased from by Evonik Industries (Essen, Germany). The chemical structures of all silanes are illustrated in Figure 1. HNT was mined and imported by Imerys Tableware Asia Limited, a New Zealand-based company, The compositions of HNT are 49.5% SiO_2_, 35.5% Al_2_O_3_, 0.29% Fe_2_O_3_ 0.09% TiO_2_ (0.09%), and a small amount of CaO, MgO, K_2_O, and Na_2_O. HNT was used as received. Global Chemical Co., Ltd. (Samut Prakan, Thailand) supplied ZnO and stearic acid. N-cyclohexyl-2-benzothiazole sulfenamide (CBS) was purchased from Flexsys America L.P. (Akron, OH, USA). Sulfur was provided by Siam Chemical Co., Ltd. (Samut Prakan, Thailand).

### 2.2. Rubber Compounding

The materials used to produce the NR/HNT composites are listed in Table 1. NR with 10 phr of HNT, an individual silane, and the other additives except for CBS and sulfur were compounded in a Brabender^®^ Plasti-Corder^®^ Lab-Station (Brabender GmbH & Co. KG, Duisburg, Germany) at a rotor speed of 60 rpm. The starting temperature of mixing was 110 °C and reached up to 150 °C before dumping. CBS and sulfur were then added to the compound in a two-roll mill. Finally, the compounds were compression-molded based on the temperature and curing time obtained from the rheometric result. The composites filled with silanized HNT were labeled based on the abbreviations of silanes such as TESPT, APTES, AEAPTMS, and VTMS whereas the control treatment was for the composite without silane.

### 2.3. Characterization and Testing

Curing characteristics were carried out using an MDR (Rheoline, Mini MDR Lite) at a temperature of 150 °C according to ASTM D5289. The results were the torque, scorch time (ts_2_), and cure time (tc_90_). Tensile tests were carried out according to ASTM D412. The dumbbell-shaped samples were prepared and tested for tensile properties using a universal tensile machine (Tinius Olsen, H10KS) at a cross-head speed of 500 mm/min. Tear strength was measured using a universal tensile machine (Tinius Olsen, H10KS) where the specimens were cut into the type C (right angle) test pieces. The tear strength was assessed following ASTM D624 with a cross-head speed of 500 mm/min. The dynamic property was examined to study the Payne effect. It was carried out using a MonTech^®^ D-RPA 3000 dynamic rubber process analyzer (RPA; MonTech Werkstoffprüfmaschinen GmbH, Buchen, Germany). The test samples were first vulcanized at 150 °C. After that, the samples were cooled to 60 °C and determined the storage modulus (*G′*) at various applied strains (e.g., 0.5% to 90%) at a fixed frequency of 10 Hz at this time. The Payne effect was used to observe filler–filler interactions using this raw G′ record. The Payne effect was calculated using Equation (1).

Payne effect = *G′_i_*–*G′_f_* (1)

where *G′_i_* and *G′_f_* were the *G′* at strains of 0.5% and 90%, consecutively. The larger the Payne effect, the higher the filler–filler interactions.

The crosslink density of the composite was determined by the equilibrium swelling method as described in ASTM D6814. The specimens were cut into a circular shape and weighed before and after immersing in toluene for 72 h. The modified Flory–Rehner equation was implemented for calculating the cross-link density (*υ*) [19]:

(2) $$\nu =\frac{1}{{2M}_{c}}$$

(3) Mc=ρ⋅V0⋅Vr13−Vr2ln1−Vr+Vr+μ⋅Vr2

where *M_c_* is the number-average molecular weight of the rubber chains between crosslinks, *µ* is the parameter for rubber–toluene interactions (*µ* = 0.42), *ρ* is the bulk density of the specimen, *V_0_* is the molar volume of the toluene (*V_0_* = 106.2 cm^3^/mol), and *V_r_* is the volume fraction in the swollen specimen, defined as follows [20]:

(4) $$V_{r}=\frac{\left(D-FT\right)\cdot \rho^{-1}}{\left(D-FT\right)\cdot \rho^{-1}+A_{0}\cdot \rho_{s}^{-1}}$$

where *T* is the weight of the specimen, *D* is the weight of the de-swollen specimen, *F* is the weight fraction of the insoluble parts, *A_0_* is the weight of the toluene absorbed in the swollen specimen, *ρ* is the density of the specimen, and *ρ_s_* is the density of the toluene (0.886 g/cm^3^).

A scanning electron microscope (Quanta 400) was applied to scan the morphology of tensile-fractured surfaces. This was carried out to see the dispersion of the HNT in the NR matrix both in the absence and presence of silanes. Gold palladium was applied to the specimen to prevent electrostatic charge buildup while examined. The behavior of strain-induced crystallization (SIC) of the composites was monitored through the synchrotron wide-angle X-ray scattering technique (WAXS) equipped with a stretching machine. The sample-to-detector distance was 115.34 mm, and the wavelength was 0.138 nm. The scattering angle was calibrated using a reference chemical, 4-bromobenzoic acid. The scattering pattern of the samples was recorded using a 165 mm-diameter CCD detector (Rayonix, SX165). The sample was stretched with certain deformation at a crosshead speed of 50 mm/min, then relaxed in such condition for 30 s. The WAXS was recorded simultaneously until the characterization was complete. The degree of crystallinity was calculated using Equation (2).

(5) $$\mathrm{Degree}\;\mathrm{of}\;\mathrm{crystallinity}\;(X_{c})=\left(\frac{A_{c}}{A_{c}+A_{a}}\right)\times 100$$

where *A_c_* is the area of crystalized peaks, and *A_a_* is the area of an amorphous region, respectively.

## 3. Results and Discussion

### 3.1. Curing Characteristics

**Figure 2:**
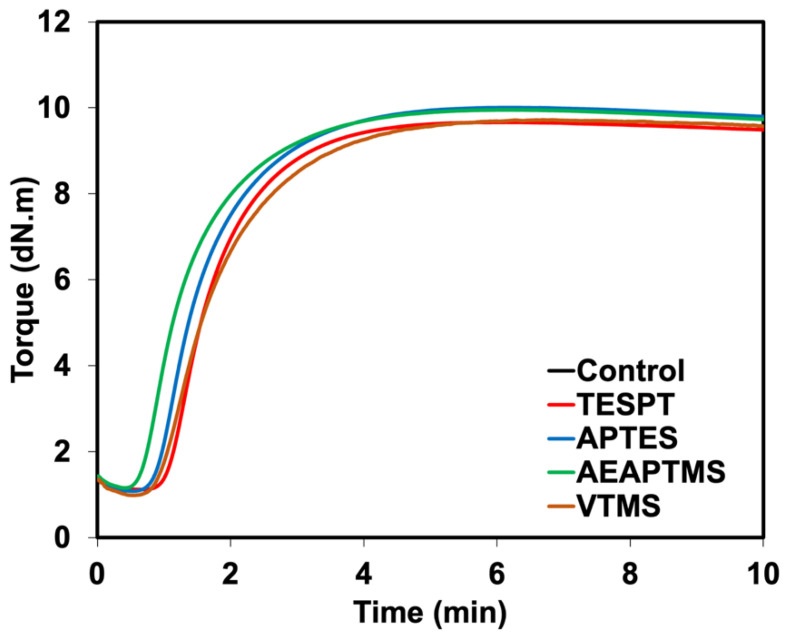
Rheometric curves of NR/HNT composites filled with and without silane coupling agents.

Figure 2 shows the rheometric curves of NR/HNT composites filled with and without silane coupling agents. The raw data extracted from rheometric curves are also listed in Table 2. There was a plateau curve when the vulcanization was complete and a small reversion at the over-curing stage. This is common behavior of NR. Treating the composites with silanes has decreased the scorch time (ts_2_) and curing time (tc_90_), as well as increased the cure rate index (CRI) than composites without silane. This happened to all silanes used for the composites. This is associated with the unique functionalities of each silane that affected the vulcanization process of rubber. For instance, the reactive sulfur atoms in TESPT can speed up the curing by yielding a reactive sulfurating agent that can form crosslinking with rubber [18]. While VTMS contains a vinyl backbone, which enables it to interact with the sulfur atoms in the rubber constituent and/or generate crosslinks through a C=C linkage [21]. Moreover, APTES and AEAPTMS contain an amine compound, which can absorb the polar sites of HNT. This has reduced the possibility of the accelerator absorbing onto the HNT surface [22]. As a result, silane decreases both the ts_2_ and tc_90_. Another possible reason may be due to the alkaline nature of amine-based silane, which increases the pH of the rubber compound. In most cases, the increased pH in rubber compounds will increase the vulcanization rate [23].

In the composite without silane, the tendency of the HNT absorbing accelerator is higher, and the silanol group of HNT is viable to form hydrogen bonds with the accelerator. This has slowed down the curing process. In the case of the composites with silanes, some silanization already took place during mixing, so there were fewer active silanol groups available for absorbing the accelerator, making the accelerator function well during vulcanization. silanes were found to raise the minimum torque (M_L_). As the compound’s viscosity influences M_L_, the interaction may also have an impact on the compound’s viscosity. The maximum torque (M_H_) and delta torque (M_H_–M_L_) of composites loaded with silanized HNT were marginally higher than those of their unsilanized counterpart. Consequently, an interaction increased these values following the addition of silanes [24]. Another explanation would be the dependence of the chemical crosslinks raised from certain silane, as the sulfur atoms, amines, and vinyl groups may influence different crosslinking mechanisms.

### 3.2. Dynamic Property

**Figure 3:**
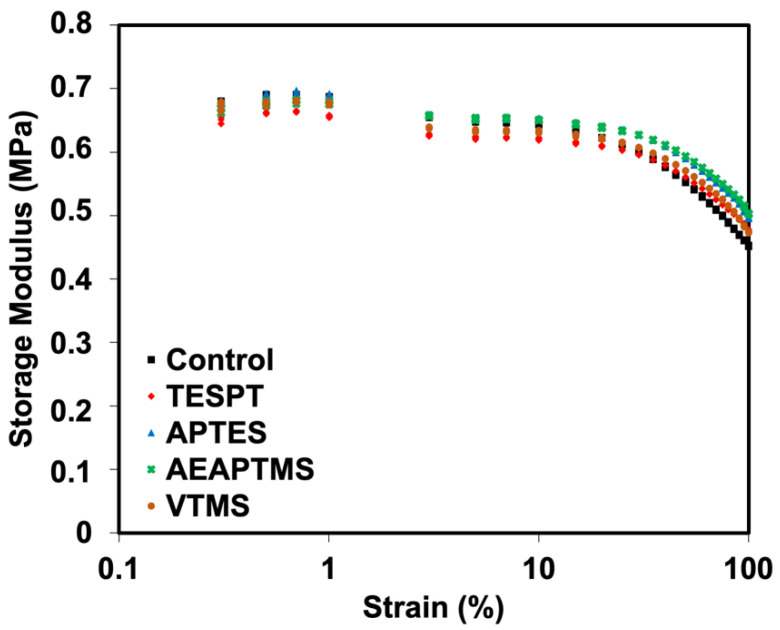
Storage modulus (*G′*) as a function of strains of NR/HNT composites filled with and without silane coupling agents.

The storage modulus (*G′*) of NR/HNT composites with and without silane coupling agents is shown in Figure 3. This technique evaluates the potential interactions within a composite [25]. All the composites showed the same tendency under low strain. *G′* declined drastically at high strain (strain > 50%). This occurs frequently with viscoelastic materials because of the rubber’s molecular stability. Treating the composites with silanes seemed to increase the *G′* value. This refers to the interactions that take place between silanized HNT and NR and result in a higher elastic response. An increase in *G′* is caused by two factors: increased interactions between polarity-matched HNT and NR and improved interfacial adhesion of HNT facilitated by silanes.

**Figure 4:**
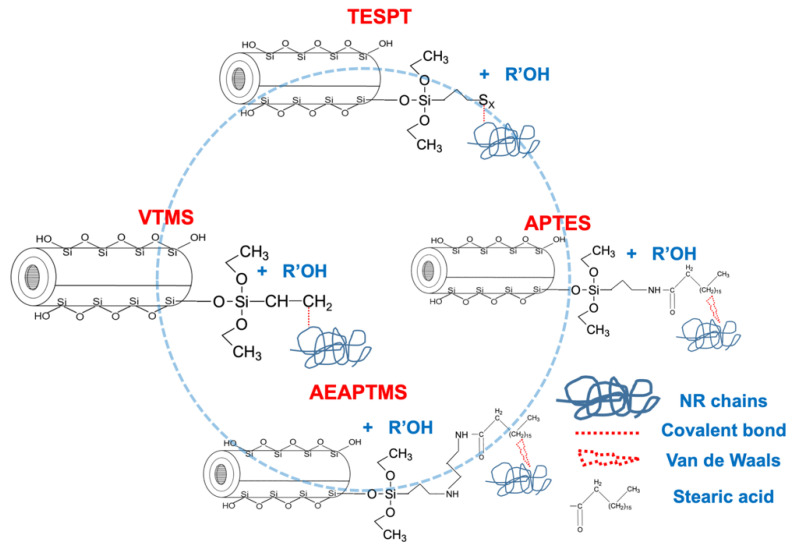
Proposed mechanisms of HNT and silane coupling agents.

The mechanisms illustrated in Figure 4 clearly show the silanization or silane to HNT reaction. The coupling or silane to NR reaction occurred differently depending on the silane type, i.e., vinyl group of VTMS or active sulfur let by the polysulfide-based silanes (TESPT), amine-group available for APTES and AEAPTMS, and the vinyl group from VTMS. Here, the coupling reaction is considered a temperature-dependent mechanism. Crosslinks were created in sulfur-based silanes (TESPT) by the sulfur released at high temperatures. At the same time, the crosslinks arose from vinyl-based silane (VTMS) through a thiol-ene reaction at the C=C bonds [19,20]. As for the amine-based silane, it was proposed by Lee et al. [26] that the amine site enables it to react with stearic acid through an amidation reaction. Such stearic acid is then reacted with ZnO to complete the vulcanization reaction.

**Figure 5:**
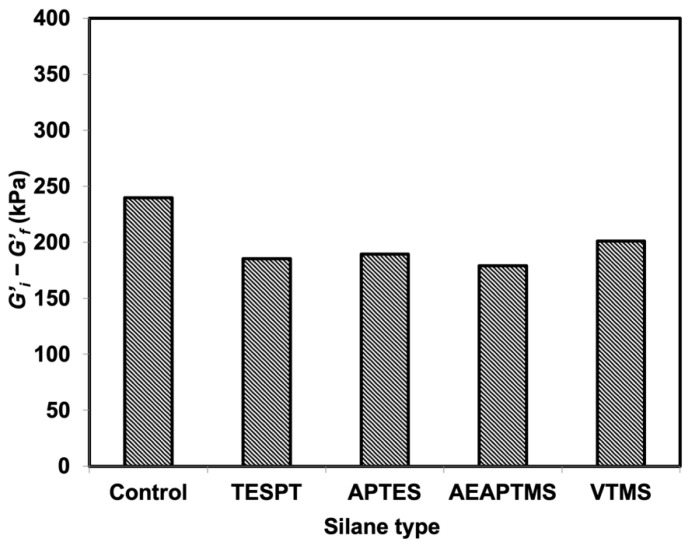
Payne effect of NR/HNT composites filled with and without silane coupling agents.

The Payne effect studies the filler–filler interactions available in a composite. The difference in *G′* between low and high strains was used to measure it. The big difference in this value indicates higher filler–filler interactions. Figure 5 shows that the filler–filler interactions were reduced after treating the composites with silanes. For example, the values were reduced from 239.64 kPa (control) to 251.80 kPa, 249.12 kPa, 214.47 kPa, and 197.19 kPa for VTMS, APTES, TESPT, and AEAPTMS, consecutively. These filler–filler interactions significantly reduced for the composites treated by TESPT and AEAPTMS. This result was used to correlate with tensile properties in the following section, showing the improvement of such properties upon treating the composites with TESPT and AEAPTMS. Moreover, AEAPTMS had the lowest value, indicating the lowest filler–filler interactions in this system. This implies that AEAPTMS is the silane of choice for NR/HNT composites to minimize the Payne effect.

**Figure 6:**
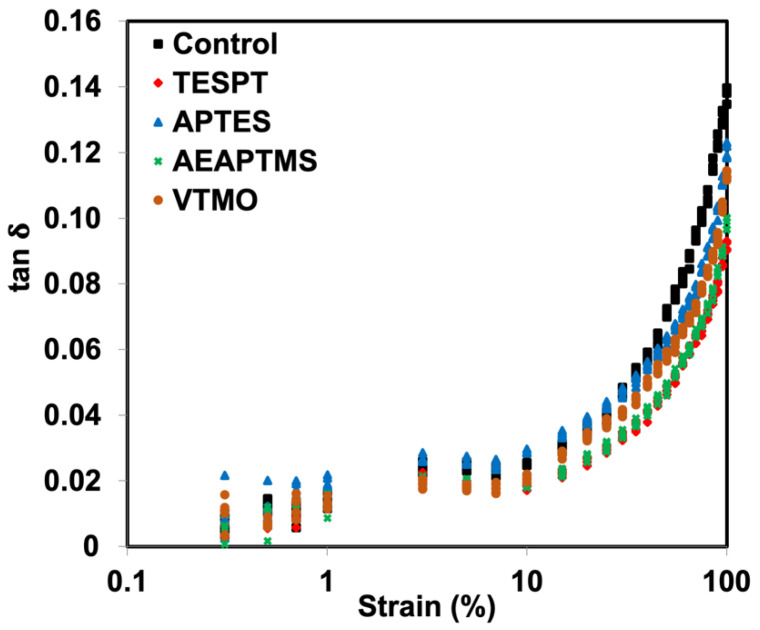
tan **δ** of NR/HNT composites filled with and without silane coupling agents.

The relationship between tan δ and strains of the NR/HNT composites with and without silanes is shown in Figure 6. A higher tan δ indicates lower elastic behavior. It is obvious that silanes have significantly influenced the damping characteristics of the composites. A lower tan δ was found for the composites containing the silanes as a coupling agent. This is a very good sign that silanes could enhance elastic response to the composite due to an improvement in rubber–filler interaction. Moreover, the lowest tan δ was observed when adding the amine-based silane. This corresponds well to the Payne effect of the composites, confirming that such an amount is sufficient to include in the composite based on NR and HNT.

### 3.3. Mechanical Properties

**Figure 7:**
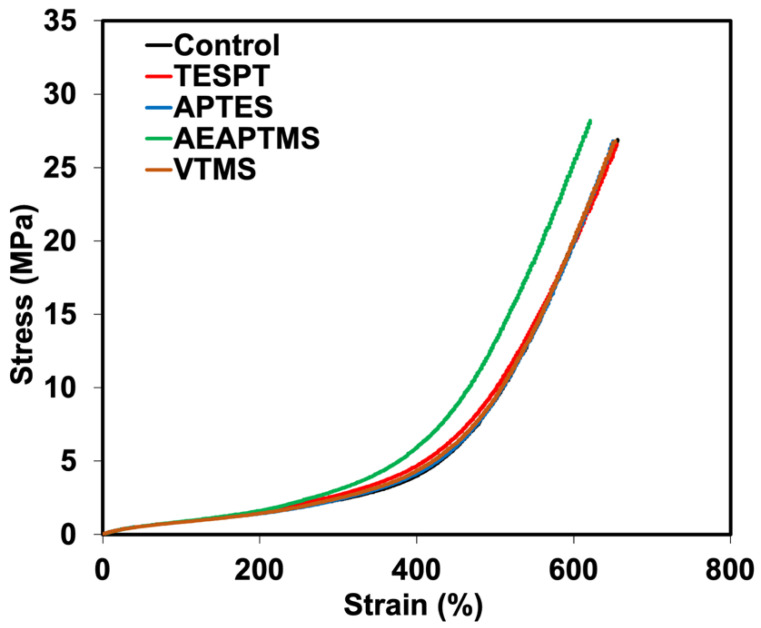
Stress-strain curves of NR/HNT composites filled with and without silane coupling agents.

The stress-strain curves of NR/HNT composites with and without silane coupling agents are shown in Figure 7. Typical stress-strain curves were seen with the occurrence of SIC. A higher stress response was found for AEAPTMS and TESPT. This suggests that the samples became stronger when these two silanes were introduced. Therefore, the improved compatibility of NR and HNT is possible. The compatibility of the NR and HNT was also confirmed by observing the area under the stress-strain curve. A broader area under the curve implies the higher toughness of rubber [27]. Composites containing AEAPTMS and TESPT exhibited improved toughness than the composite with no silane because they had a wider area under the stress-strain curve.

Tensile strength, elongation at break, and tensile modulus of NR/HNT composites with and without silanes are listed in Table 3. For the composites containing silanes, slightly greater stress at strains of 100% (M100) and 300% (M300) was observed. The highest modulus was revealed to be in the AEAPTMS. As previously mentioned, an amine-based accelerator could perform well-silanization, thereby providing composites with better rubber–filler interactions. However, although TESPT contains a certain amount of sulfur to give an extra crosslinking to NR, it may not be viable in this system. This scenario contradicts the report of Kaewsakul et al. [22], who found that TESPT provided maximum performance to the NR/silica composites due to the sulfur atoms available in its structure. However, such composites contained two modifiers, i.e., silane and ENR, which may perform differently. Consequently, an amine-based silane seems to be workable in this case.

**Figure 8:**
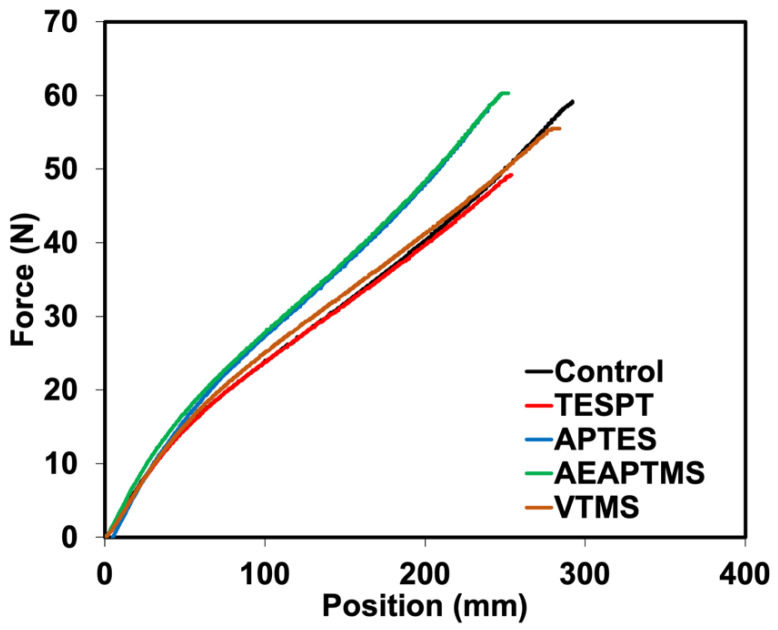
Tearing force as a function of extension of NR/HNT composites containing various content of BFA.

**Figure 9:**
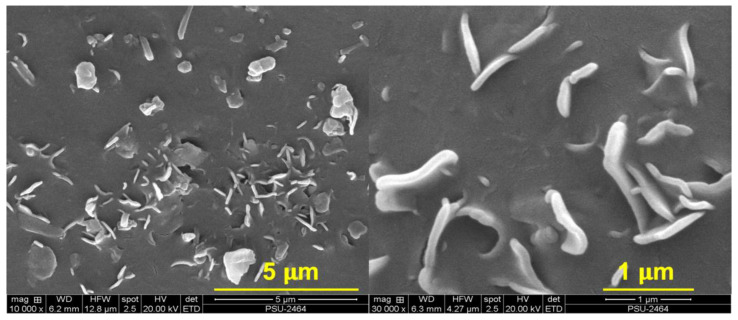
SEM images at 10,000× (**left**) and 30,000× (**right**) magnifications of NR/HNT composites.

**Figure 10:**
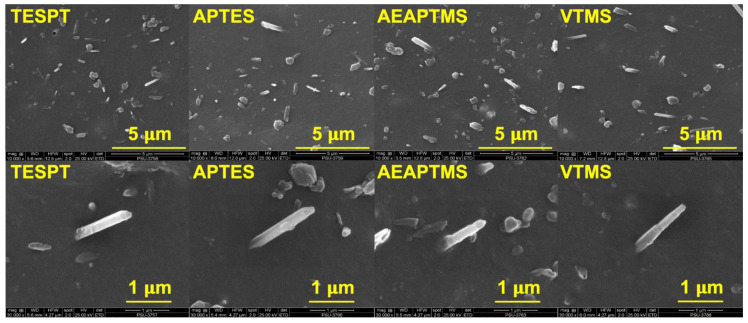
SEM images at 10,000× (**top**) and 30,000× (**bottom**) of NR/HNT composites filled with silane coupling agents.

The findings matched a drop in elongation at the break of the composites. This was associated with lower filler–filler interactions, leading to a decrease in the elasticity of the composite. The tensile strength and tear strength (see Figure 8) of the composites also increased when adding silanes, particularly for AEAPTMS. The tendency of tensile strength grew from 26.84 MPa to 28.17 MPa, while the tear strength of the composites rose from 25.49 N/mm to 27.68 N/mm. As a result, it is clear that AEAPTMS increased interfacial adhesion through specific chemical interactions with HNT. Evidence of such enhancement was already stated in previous sections. The SEM micrographs of composites with different silane coupling agents are displayed in Figure 9 and Figure 10. Figure 9 shows the SEM images of the composite without silane. It was seen that there was a high tendency for filler agglomeration to appear in the specimen. In Figure 10, distinct tubular shapes of HNT have evenly dispersed throughout the matrix thanks to the application of silanes, which has considerably increased filler dispersion. This fits the mechanical characteristics discovered in the prior explanation. This might have impacted the composite’s tensile and tear strengths.

### 3.4. Wide Angle X-Ray Scattering

**Figure 11:**
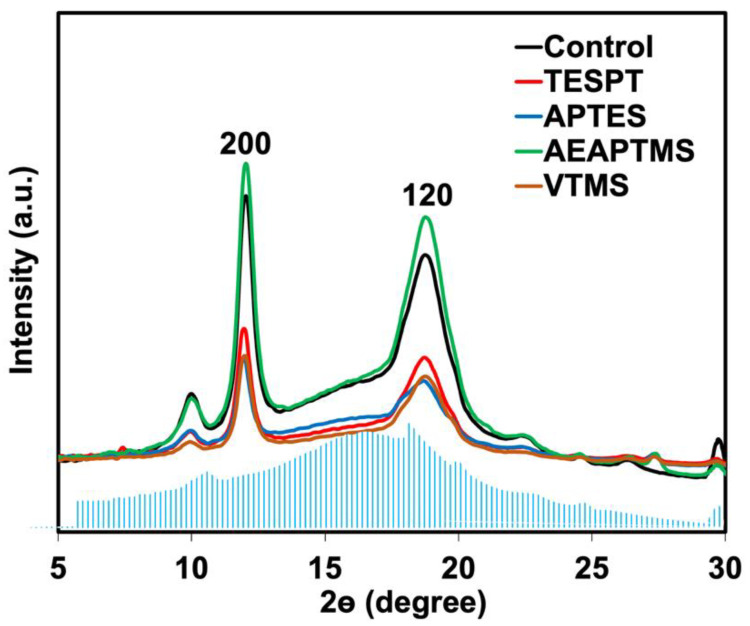
1D WAXS patterns of unstretched (blue highlight) and 400% stretched NR/HNT composites filled with and without silane coupling agents.

**Figure 12:**
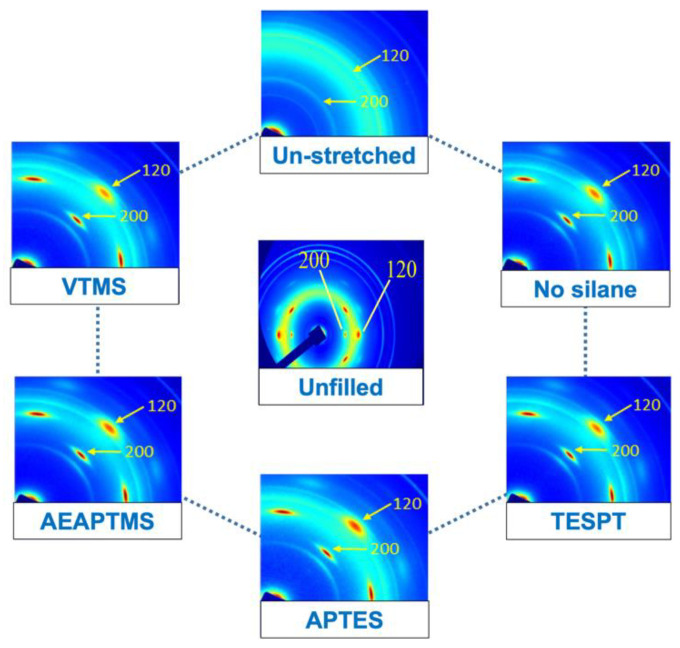
2D WAXS images of unstretched and 400% stretched NR and NR/HNT composites filled with and without silane coupling agents.

It is well-recognized that strain-induced crystallization (SIC) is behind the excellent mechanical properties of NR [28,29]. Adding filler to NR can also improve the ability of SIC, especially in a compatible composite. It was expected that the SIC of the composites could be improved by adding silane coupling agents. Therefore, WAXS was analyzed to monitor the development of SIC. One-dimensional X-ray diffraction (1D-WAXS) and two-dimensional (2D-WAXS) at 400% strain of the composites are shown in Figure 11 and Figure 12. The blue highlight in the mountain area embedded in the Figure showed the XRD pattern of the unstretched sample. It indicates the amorphous pattern of NR before stretching. This shows that no crystallization occurred at this stage. Upon applying the strain, the crystal diffraction peaks are shown by reflections on the 200 and 120 planes of NR at 2θ angles of 12.0–12.5° and 18.0° [30]. The crystallinity at 200 and 120 planes was clear for AEAPTMS, this again verified that using this silane has given remarkable properties to the NR/HNT composite, making it a silane of choice for the composite.

These two peaks became more intense with AEAPTMS. That means NR has more crystallization in the presence of silanes. The 2D-WAXS image shows clear evidence of a crystallization point. Crystallized spots of stretched samples were observed compared to the unstretched sample. Considering unfilled and filled NR composites, it also appeared that the crystallized spots became clearer for the filled NR. The reasons behind such a finding were previously reported in the literature regarding the SIC of NR facilitated by the presence of fillers [11,12]. When the silane coupling agents were used, the crystallization point became clearer, and the amorphous part (mountain area) became lighter. This demonstrated that silane affected the crystallization of NR. Improved interaction between the NR and HNT resulted in faster molecular chain alignment and affected the ability to crystallize the NR. The presence of an interaction in rubber is a behavior similar to cross-linking. In most studies, it is said that crystallization in rubber can be activated by crosslinking in rubber. Spratte et al. [31] also reported on the SIC of silica-filled NR in the presence of a silane coupling agent. The silanization and coupling interactions/reactions taking place in their composite can limit the movement of the rubber chain, which then increases the ability to crystallize. Such interactions/reactions reduce the onset of crystallization as the crystallization starts to occur at a very low strain. The results from their WAXS profiles also correlated with the tensile strength of the composites.

**Figure 13:**
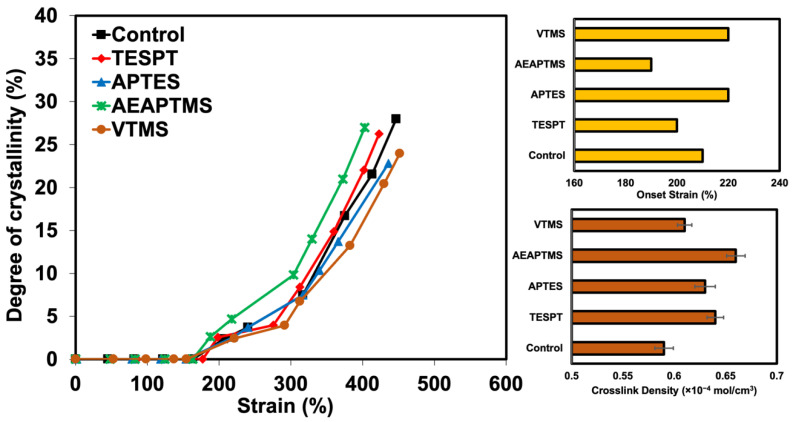
Degree of crystallinity as a function of applied strains together with onset strains and corresponding crosslink density of NR/HNT composites filled with and without silane coupling agents.

The degree of crystallinity (*X_c_*) as a function of strains can be calculated from the intensity of the subpeak area from the crystalline reflections on the 200 and 120 planes. Figure 13 shows the change in crystallization degree at different strain levels. It was found that the crystallization increases with increasing deformation. Also, the crystallization increases with the use of AEAPTMS and TESPT. Better rubber–filler interactions assisted by these two silanes acted as a hook to gather the surrounding molecular chains. This ability has promoted more crystallization upon stretching. It was also observed that the onset strain was reduced for these two silanes too. This clearly indicates that AEAPTMS and TESPT assist in the crystallization of NR, which corresponds well with the dynamic study reported in the previous section. AEAPTMS and TESPT can establish more interactions between NR and HNT through certain chemical interactions. This results in more efficient stress transfer from the rubber to the HNT, which is an important point in the formation of crystals (Crystallized point) and also allows the molecular chains of NR to be more organized. However, the crystals formed were too large.

The onset for SIC with AEAPTMS and TESPT was observed to be lower as a function of urea content. The interaction in the presence of silanes can help pull the surrounding molecular chains and speed up the crystallization process. Ozbas et al. [32] compared the SIC of graphene and CB-filled composites. They found that the onset of SIC occurs at significantly lower strains for graphene-filled NR samples compared with CB-filled NR even at low loadings. Moreover, additional interaction in the rubber network is also responsible for either accelerating or slowing down the crystallization rate depending on the rubber matrix chemical crosslink density. Candau et al. [25] and Ozbas et al. [32] further emphasized that the rubber–filler interactions may fasten the SIC at low or high crosslink density. This is because high crosslinking may interfere with the chain orientation and reduce the SIC. Therefore, the crosslink density of this composite was also reported and shown in Figure 13. It can be seen that even the crosslink density observed was slightly increased by silanes. It is still sufficient to help develop the SIC. Consequently, it can be said that the change in SIC was promoted by rubber–filler interactions.

**Figure 14:**
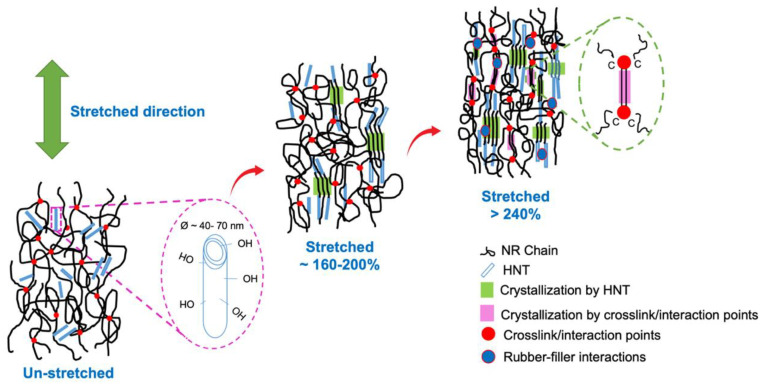
Schematic illustration of SIC in the NR/HNT composites filled with silane coupling agents.

Based on these findings, Figure 14 schematically discusses a relationship between strain-induced crystallization and, correspondingly, rubber–filler interactions. Two stages of crystallization are depicted in this diagram. The initial stage occurs at a lower strain of roughly 160–200%. HNT may entangle well with the NR chains due to the better interfacial adhesion facilitated by silanes. HNT is oriented and aligned along the stretching direction of the specimen. This has caused the NR to perform the SIC, whereby the crystallinity rises in correlation with the orientation of the HNT. The NR chains subsequently reorganize and crystallize as a result. Second, at stresses of more than 240%, the NR matrix’s crystallinity sharply rises as a result of the cooperative crystallization of NR and HNT, along with the contribution of the silane coupling agents, in this case, AEAPTMS and TESPT. The surrounding molecular chains are pulled in large part by the hydrogen bonds created by the presence of the silane coupling agent. As a result, at greater strains, a noticeable increase in crystallization is shown, which is consistent with earlier findings in the stress-strain behaviors and WAXS profiles.

## 4. Conclusions

The overall characteristics of NR/HNT composites were clearly improved by introducing silanes. Regardless of how they act, using silanes has increased modulus, tensile strength, and tear strength. This was due to the formation of silanization and coupling reactions within the composites. Using AEAPTMS as silane was the most viable for NR/HNT composites. The result from the Payne effect proved that using AEAPTMS gives the lowest filler–filler interactions. SIC is another key performance that can be used to evaluate the prepared composites. It is clear that a significant change in the strain upturn was found for AEAPTMS as silane. The onset strain of such a composite was at an earlier strain during stretching. This indicated faster crystallization due to better interfacial interactions within the composites. The surrounding molecular chains were drawn to silane like a hook, increasing the crystallinity of the composites. All of the supporting data correlated well with earlier findings in the stress-strain behavior and WAXS profiles.

## Figures and Tables

**Table 1:** The formulation used for compounding NR/HNT composites filled with and without silane coupling agents.

Ingredients	Amount (phr)				
	Control	TESPT	APTES	AEAPTMS	VTMS
RSS 3	100	100	100	100	100
Zinc oxide	5	5	5	5	5
Stearic acid	1	1	1	1	1
HNT	10	10	10	10	10
Silane *	-	0.5	0.4	0.4	0.3
CBS	2	2	2	2	2
Sulfur	2	2	2	2	2

* The content of silane is based on molar equivalent of silane to HNT.

**Table 2:** Raw data extracted from the rheometric curves of NR/HNT composites filled with and without silane coupling agents.

Silane Type	M_L_ (dN.m)	M_H_ (dN.m)	M_H_-M_L_ (dN.m)	ts_1_ (Min)	tc_90_ (Min)	CRI (Min^−1^)
Control	1.12	9.66	8.54	1.15	2.99	32.29
TESPT	1.14	9.61	8.47	1.24	2.84	33.97
APTES	1.08	10.01	8.93	0.98	3.02	32.13
AEAPTMS	1.15	9.95	8.80	0.76	2.84	34.45
VTMS	0.98	9.72	8.74	1.04	3.33	28.99

**Table 3:** Raw data extracted from the rheometric curves of NR/HNT composites filled with and without silane coupling agents.

Silane Type	Tensile Strength (MPa)	Elongation at Break (%)	M100 (MPa)	M300 (MPa)
Control	26.84 ± 0.36	656 ± 19	0.85 ± 0.01	2.37 ± 0.02
TESPT	26.57 ± 0.52	655 ± 16	0.87 ± 0.02	2.67 ± 0.12
APTES	26.81 ± 0.45	650 ± 6	0.86 ± 0.48	2.40 ± 0.49
AEAPTMS	28.17 ± 0.19	621 ± 19	0.90 ± 0.01	3.03 ± 0.18
VTMS	26.84 ± 0.45	653 ± 9	0.84 ± 0.02	2.50 ± 0.17

## Data Availability

The data presented in this study are available on request from the corresponding author.
